# Efficacy of Sorafenib-Based Therapies for Non-Small Cell Lung Cancer

**DOI:** 10.3390/medsci12020020

**Published:** 2024-04-07

**Authors:** Morgann Hendrixson, Yevgeniy Gladkiy, Anita Thyagarajan, Ravi P. Sahu

**Affiliations:** 1 Boonshoft School of Medicine, Wright State University, Dayton, OH 45435, USA; hendrixson.7@wright.edu (M.H.); gladkiy.2@wright.edu (Y.G.); 2 Department of Pharmacology and Toxicology, Boonshoft School of Medicine, Wright State University, Dayton, OH 45435, USA; anita.thyagarajan@wright.edu

**Keywords:** sorafenib, targeted therapy, non-small cell lung cancer, cell signaling pathway

## Abstract

Lung cancer remains the leading cause of cancer-related deaths, with a poor prognosis. Of the two types, non-small cell lung cancer (NSCLC) is the major and most prevalent type and associated with low response rates to the current treatment options. Sorafenib, a multitargeted tyrosine kinase inhibitor used for various malignancies, gained attention for its potential efficacy in NSCLC. This review paper focuses on the findings of recent in vitro, in vivo, and clinical studies regarding the efficacy of sorafenib. Overall, sorafenib has shown definitive therapeutic potential in NSCLC cell lines, xenografts, and human subjects. Novel approaches to sorafenib delivery may improve its efficacy and should be the focus of further studies.

## 1. Introduction

Globally, lung cancer has the highest mortality of any cancer type and the second highest incidence rate, following only slightly behind breast cancer [1,2]. Although lung cancer 5-year survival rates have been improving since 1975 in the United States, the 5-year survival rate remains lower than 30% [3,4]. Lung cancer is divided into two main subtypes—small cell lung cancer (SCLC) and non-small cell lung cancer (NSCLC), the latter of which makes up nearly 85% of cases [5,6]. Advancements in cancer therapy have improved the prognosis of NSCLC. Previously, NSCLC was treated primarily with platinum-containing drugs, such as carboplatin or cisplatin. These regimens, often combined with other cytotoxic agents, aimed at impeding the rapid proliferation of cancer cells. While they provided an initial efficacy, the associated toxicities and limited overall survival prompted a shift to more targeted and tolerable approaches [7,8]. Immunotherapy and targeted therapies to improve overall survival have emerged as prominent choices for intervention in patients with NSCLC [9,10]. Immune checkpoint inhibitors, such as nivolumab, atezolizumab, and pembrolizumab, have shown remarkable success in subsets of NSCLC patients [11,12,13,14]. Additionally, targeted therapies against specific mutations such as EGFR and ALK have shown promising efficacy [15,16] However, with the diverse molecular profiles of NSCLC and variations in treatment response, resistance, and tolerability, research continues to evaluate the efficacy of additional monotherapy and dual-therapy approaches for patients with advanced NSCLC. 

In this review, we will focus on a specific agent, sorafenib, in the treatment of NSCLC. The mechanisms of action and resistance will be discussed, followed by an in-depth discussion of the existing literature regarding sorafenib’s efficacy in NSCLC. We will summarize the findings of recent in vitro, in vivo, and clinical studies. Lastly, we will discuss our overall impression of sorafenib’s efficacy based on the existing literature and future directions for studies regarding sorafenib. 

## 2. Mechanisms of Action and Resistance of Sorafenib 

**Figure 1:**
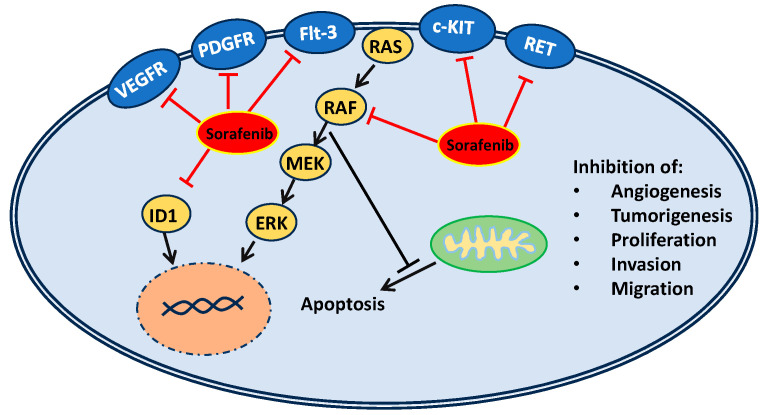
Schematic representation of the mechanisms of sorafenib action.

Sorafenib (BAY 43-9006, Nexavar) is an oral, FDA-approved multitargeted tyrosine kinase inhibitor that has been shown to increase survival in renal cell, hepatocellular, and thyroid cancer. [17,18,19]. Initially, sorafenib was identified as a potent RAS/RAF/MEK/ERK pathway inhibitor with additional activity against angiogenesis (i.e., vascular endothelial growth factor receptor [VEGFR-2/3], platelet-derived growth factor receptor [PDGFR]), and tumorigenesis (FMS-like tyrosine kinase 3 [Flt-3], a type III receptor tyrosine kinase [c-KIT], rearranged during transfection [RET]) [20,21,22]. Recently, inhibitor of differentiation 1 (ID1) expression has been shown to be inhibited in NSCLC cells by sorafenib, impacting cell proliferation, invasion, and migration [23]. Following sorafenib’s approval, other tyrosine kinase inhibitors (TKIs) have been shown to improve treatment outcomes of the aforementioned diseases, but it remains the only approved therapy for hepatocellular carcinoma (HCC) and is commonly used in treatment of solid tumors [24]. Promising biomarkers such as a high KIT concentration or low hepatocyte growth factor (HGF) have indicated enhanced survival, but none have been validated for clinical use to show responsiveness to sorafenib [24]. The schematic representation of sorafenib’s mechanisms of action is given in Figure 1.

**Figure 2:**
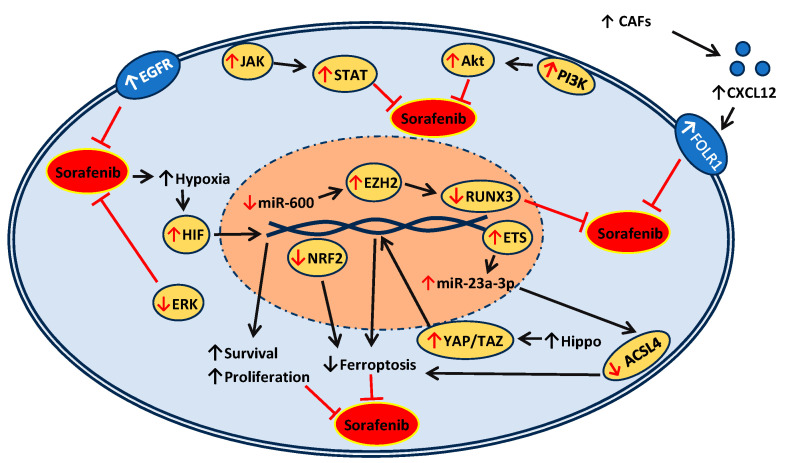
Schematic representation of the mechanisms of sorafenib resistance.

The exact mechanisms of resistance to sorafenib remain unclear. However, resistance to sorafenib can be self-imposed, as it is known to intensify tumor hypoxia, which paradoxically facilitates cell survival and propagation of the tumor [25,26,27]. Besides, epidermal growth factor receptor (EGFR) overexpression has also been shown to inhibit the antitumor effect but downregulation of its downstream signaling molecule, the extracellular signal-regulated kinase (ERK) of the Ras/Raf/MEK/ERK axis, has been predicted to overcome sorafenib resistance [27]. In addition, activation of the Phosphatidylinositol 3-kinase/protein kinase B (PI3K/Akt) pathway and abnormal changes in the Janus kinase/signal transducers and activators of transcription (JAK/STAT) pathway, such as a high expression of STAT3, have also shown to induce sorafenib resistance [27]. Moreover, other resistance mechanisms, such as the accumulation of autophagosomes, epithelial–mesenchymal transition, hypoxia-inducible factor, and histone modification, as well as others, are discussed by Zhu and colleagues [27]. Furthermore, the downregulation of ferroptosis has been shown to increase resistance to sorafenib [28,29,30]. The schematic representation of sorafenib’s mechanisms of resistance is given in Figure 2.

In the Hippo/Yes-associated protein (YAP) signaling axis, the dysregulation of Hippo results in tumorigenesis, promoting YAP/TAZ, which further induces proliferation and chemoresistance to sorafenib through the inhibition of ferroptosis [28,30]. Further studies in HCC showed that sorafenib-resistant cells increased miR-23a-3p transcription in an ETS Proto-Oncogene 1 (ETS1)-dependent manner, and targeting the key positive regulator of ferroptosis, acyl-CoA synthetase long chain family member 4 (ACSL4), has been shown to overcome sorafenib resistance. A complete review of ferroptosis-related sorafenib resistance can be reviewed in Guo et al. and Wang et al. [28,30]. Furthermore, other HCC mechanisms of resistance include the activation of cancer-associated fibroblasts (CAFs) via the CXCL12/FOLR1 axis [31]. Overlapping with clear cell renal cell carcinoma (ccRCC), the nuclear factor erythroid 2-related factor 2 (NRF2)-dependent inhibition of ferroptosis also induces sorafenib resistance [32]. In a recent in vitro breast cancer study of miR-600, a low expression of miR-600, the inhibition of its downstream target enhancer of zeste homolog 2 (EZH2), an oncogenic histone methyltransferase, and EZH2’s inhibition of tumor suppressor gene runt-related transcription factor 3 (RUNX3), have been shown to cause sorafenib resistance [33].

## 3. Studies of Sorafenib Efficacy in NSCLC 

### 3.1. In Vitro and In Vivo Studies of Monotherapy and Dual-Therapy Approaches Involving Sorafenib 

The first in vitro study of note assessed the efficacy of sorafenib in combination with gemcitabine or pemetrexed in NSCLC cell lines with KRAS mutations [34]. Monotherapy with sorafenib, gemcitabine, and pemetrexed exhibited inhibitory effects on KRAS-mutated A549 NSCLC cells. However, combination therapy with sorafenib + gemcitabine yielded greater anti-proliferative effects than the sum of the individual effects added together, with a combination index of 0.86. Importantly, the combination therapy of sorafenib and pemetrexed displayed greater inhibitory synergistic effects (CI 0.63) than the sorafenib/gemcitabine therapy (CI 0.86) [34]. The summary of in vitro studies demonstrating sorafenib combination indexes with other drugs is given in Table 1. 

More recently, a combination of sorafenib + gemcitabine was studied using in vitro (A549, H1975, H1650 cell lines) and in vivo mice xenografts for NSCLC [35]. The results of this in vitro study indicated synergism with this combination therapy, similar to the findings of Li et al. above (CI 0.65). However, this study also demonstrated the synergistic activity of sorafenib in vivo, as evidenced by a decreased tumor growth and tumor weight in mice treated with this dual therapy. In addition, it was determined that sorafenib + gemcitabine therapy exerted anti-migration and anti-invasion effects by inhibiting epithelial-to-mesenchymal transition (EMT). In summary, this study suggests that combination therapy with sorafenib + gemcitabine may provide an effective way to inhibit metastasis via the targeting of EMT and provide better outcomes in patients with advanced NSCLC [35]. 

Kutkowska et al. also found inhibitory synergistic effects when using sorafenib in combination with betulinic acid in three NSCLC lines with different KRAS mutations: A549 (KRASG12S), H358 (KRASG12C), and A427 (KRASG12D) [36]. Interestingly, greater apoptotic effects were observed at even very low concentrations of combination therapy, compared to the monotherapy of either agent. Additionally, a Western blot analysis of A549 cells treated with sorafenib + betulinic acid revealed an inhibition of the Akt pathway but not the MAPK pathway. In summary, adding betulinic acid to sorafenib may provide greater antitumor effects in NSCLC, while also allowing lower concentrations of each agent to be used in order to reduce drug-related toxicities. However, further in vivo and clinical studies are warranted [36]. 

Another study found that fingolimod (FTY720), a sphingosine analog, in combination with sorafenib, exhibited significant cytotoxic effects on EGFR wild-type A549 NSCLC cells [37]. The combination index was 0.74, indicating the synergistic effect of the dual therapy, compared to the monotherapy of either agent. In addition, cell cycle analysis of A549 cells treated with sorafenib and fingolimod combined therapy revealed an increased G_0_/G_1_ and G_2_/M cell cycle arrest compared to non-treated cells, indicating the cytostatic effects of the combined drug regimen [37]. 

All three of these recent in vitro studies reported combination indices (CIs) to represent the relationship shared between the drugs used for the combination therapies in comparison to their individual counterparts. Regarding these CI values, a value less than 1 indicated a synergistic relationship between the two drugs in the combination, a CI equal to 1 represented an additive relationship between the dual therapy agents, and a CI > 1 indicated the antagonism of the two drugs. All three of the studies mentioned here had CI values < 1, indicating that each of the combined therapy regimens exerted synergism. Values closer to zero represented greater synergism. Therefore, the greatest synergism in the A549 cell line was seen with the sorafenib + pemetrexed combination. However, the A429 cell line studied in Kutkowska et al. had the greatest overall synergistic effect, with a CI of 0.497 (Table 1) [35,36,37]. 

Chen et al. conducted in vitro and in vivo studies involving carboxyamidotriazole (CAI) and sorafenib combination therapy in Lewis lung carcinoma (LLC), A549, and H1975 NSCLC cells and mice models [38]. CI values were used to determine the relationship between sorafenib and CAI at different drug concentrations (sorafenib 0.5–10 µM; CAI 1–20 µM) and three different time points (24, 48, 72 h). The results revealed CI values < 1 at all time points and combination drug concentrations in LLC cells, thus exhibiting the fully synergistic relationship of the combination therapy in these cells. A549 cells had CI values < 1 for almost all time points and drug combinations with few exceptions, suggesting a synergistic relationship. However, the H1975 cell line exhibited a CI < 1 for low combination therapy concentrations but values > 1 at higher concentrations, suggesting a lack of efficacy at high concentrations but synergism at lower concentrations of the dual therapy. It is important to note that the A549 cell line has an EGFR wild-type with KRAS-mutant, whereas the H1975 cell line has an EGFR double mutant with KRAS wild-type. Therefore, sorafenib/CAI therapy may be more effective in individuals with KRAS mutations and without the double-mutant EGFR associated with the H1975 cell line [38]. However, at low concentrations, even those with the EGFR mutation seen in the H1975 cells may benefit. In terms of the mice experiments, it was determined that combination therapy with sorafenib + CAI significantly decreases tumor growth. The efficacies of this combination therapy and high-dose sorafenib alone were similar. However, cancer-related cachexia was less severe in mice treated with the combination therapy, therefore making it a more optimal option [38] (Table 1). 

Sorafenib was also found to have a synergistic relationship with a PI3K/mTOR inhibitor synthesized by Wang and colleagues [39]. This novel inhibitor, known as S1, in combination with sorafenib, was found to inhibit cell proliferation, migration, and cell invasion in cell lines A549, H157, and 95D more than the sum of the agents used independently. In vivo studies revealed enhanced tumor growth suppression when using this combination therapy in A549 NSCLC xenografts and improved survival times. In addition, Western blot analyses revealed that sorafenib exerted a significant inhibition of ERK phosphorylation involved in the Ras/Raf/MEK/ERK pathway. However, S1 exerted a significant inhibition of the S6 phosphorylation involved in the PI3K/Akt/mammalian target of the rapamycin (mTOR) pathway. Given these findings, these researchers concluded that the synergism seen with the combination therapy of sorafenib and S1 may be due to this differential phosphorylation and may provide a novel approach to treating NSCLC [39]. 

Furthermore, an erastin + sorafenib combination has been studied by Li and colleagues in NSCLC cell lines A549 and H1299, as well as in vivo in mice xenograft models following cisplatin treatment failure. This study revealed that erastin + sorafenib dual therapy effectively induced ferroptosis in cisplatin-resistant cells, as well as in the xenograft model, thus suggesting the potential utility of this combination for NSCLC [40]. 

### 3.2. In Vitro and In Vivo Studies with Novel Approaches to Sorafenib Delivery 

Nanotechnology is rapidly gaining popularity as a mechanism for drug delivery in cancer patients. Nanocarriers have proven efficacious in improving delivery to specific locations, as well as improving the absorption of pharmacologic agents used in cancer treatment [41]. Zhong and colleagues specifically studied the delivery of sorafenib (and crizotinib) within polymeric nanoparticles [42]. These researchers found that, in the xenograft lung cancer model, delivery of these drugs within the nanoparticles improved survival and produced fewer side effects than the drugs given intravenously without the encapsulation of the nanoparticles. In addition, the in vitro studies showed a quicker absorption of the drugs when delivered via nanoparticles [42]. 

Along similar lines, Shukla and colleagues studied sorafenib delivery within inhalable nanocarriers for NSCLC in vivo and in vitro. These researchers found that delivery with inhalable carriers improved antitumor activity, compared to traditional sorafenib delivery. The inhalable nanocarriers were found to be stable for up to 30 days at 4 °C and 25 °C but were only viable up to 15 days at physiologic temperatures (~37 °C). In addition, the cationic coating of the nanoparticles enhanced the cellular uptake of sorafenib, compared to nanoparticles without the positively charged coating [43]. 

Furthermore, sorafenib delivery within a liposomal dry powder inhaler was studied in vitro using NSCLC by Patel and colleagues. In this study, the liposomal inhaled formulation had a greater encapsulation efficiency and drug content, low density, and enhanced lung absorption than the traditional delivery method of sorafenib. Moreover, delivery of sorafenib with the inhaler was found to follow a biphasic release pattern, with a burst release occurring within 6 h, followed by a sustained release for up to 72 h. Given these findings, sorafenib delivery within these liposomal inhalers may provide a unique mechanism to decrease sorafenib dosages and/or frequencies for patients requiring this therapy [44,45]. 

In addition, our recent studies have implicated the involvement of tumor-secreted large extracellular vesicles, known as microvesicle particles (MVPs) or miRNA-149-5p, in modulating the cellular responses of chemotherapy or targeted therapy [46,47,48]. In particular, these MVPs generated in response to chemotherapy or targeted therapies carry a potent phospholipid mediator, platelet-activating factor-receptor (PAFR) agonist, which has been shown to be involved in augmenting lung cancer growth and metastasis in a PAFR-dependent manner [49]. It is of importance to note that there is an emerging interest in exploring the efficacy of exosomes and MVP-based therapeutics for cancer intervention [50].

### 3.3. Clinical Studies Involving Sorafenib 

Multiple clinical trials have been conducted in the past several years to assess the efficacy of sorafenib in NSCLC. The first was a phase II trial known as the BATTLE trial (Biomarker-integrated Approaches of Targeted Therapy for Lung Cancer Elimination) [9,51]. In this clinical trial, sorafenib efficacy in advanced NSCLC was evaluated using disease control rates (DCRs), overall survival (OS), and progression-free survival (PFS). After eight weeks of treatment with 400 mg sorafenib two times daily, efficacy results were collected with the aforementioned measures in eligible patients. For these 98 patients, the DCR was 58.2%, indicating that 57 patients obtained disease stability. The median PFS was found to be 2.83 months, and the median OS was 8.48 months. Regarding biomarker analysis, the DCR was higher among individuals with wild-type EGFR tumors (64.2%) compared to EGFR-mutant tumors (23.1%). However, the comparison of PFS was not significant. It is also important to note that the DCR and PFS of patients with EGFR FISH-negative tumors were significantly higher compared to FISH-positive tumors. Furthermore, a sorafenib sensitivity signature (SSS), created from NSCLC wild-type EGFR cell lines, was used in vitro in patient tumor biopsy samples. After 8 weeks, the PFS of EGFR wild-type tumors treated with a high SSS was greater than in those treated with a low SSS. However, the difference in DCRs was not significant (Table 2) [9,51]. With these findings that the SSS may help predict which patients with NSCLC wild-type EGFR tumors may benefit most, these researchers continued to study this signature in the BATTLE-2 trial. Importantly, the researchers noted that individual EGFR biomarkers had greater predictive value than the biomarker groups used in this study, therefore creating a limitation later addressed in their BATTLE-2 study [52].

The phase II BATTLE-2 trial focused primarily on KRAS-mutant NSCLC tumors and was conducted with the goal of determining biomarkers for increased the efficacy of targeted treatments and the efficacy of an SSS described in the BATTLE study. However, the use of an SSS in this study did not impact the DCR or PFS in KRAS-mutated tumors, thus prompting the need for further research on the resistance mechanisms of sorafenib [52].

Around the same time that the BATTLE trial results were published, results from a phase III trial known as the MISSION trial were also published. The MISSION trial evaluated single-agent third-to-fourth line sorafenib therapy in patients with NSCLC, compared with a matching placebo. Although overall survival was similar in patients treated with the placebo and sorafenib, the PFS increased in individuals treated with sorafenib (2.8 months) compared to the placebo (1.4 months). Biomarker analyses revealed that patients with EGFR mutations who received sorafenib had a significantly higher median PFS (2.7 vs. 1.4 months) and higher median OS (13.9 vs. 6.5 months) compared to patients who received the placebo. Patients with KRAS mutations who received sorafenib also displayed a longer PFS but no significant difference in OS (Table 3) [53].

Several other phase II studies have been conducted to assess the efficacy of sorafenib in advanced NSCLC (summary given in Table 4 and Table 5). Dingemans and colleagues evaluated the utility of sorafenib in patients with KRAS-mutated NSCLC who were pretreated with platinum-based therapies. Following six weeks of sorafenib therapy, the DCR was 52.6%. The PFS and OS were 2.3 months and 5.3 months, respectively. The type of KRAS mutation did not significantly affect the PFS or OS. These findings of a DCR > 50% suggest the utility of sorafenib in some patients with KRAS-mutated NSCLC. However, with the relatively low PFS and OS and the insignificance of the type of KRAS mutation, the relationship remains elusive [54].

More recently, Spigel and colleagues conducted a phase II clinical study comparing monotherapy sorafenib to dual sorafenib + erlotinib in patients with NSCLC, who had previous improvement with erlotinib but experienced current progression on the erlotinib monotherapy [55]. The PFS of patients treated with sorafenib + erlotinib was longer (3.1 months) compared with sorafenib monotherapy (1.9 months), whereas, the OS of patients treated with sorafenib + erlotinib (8.9 months) was shorter than that of patients who received monotherapy (11.9 months). However, neither of these results was statistically significant (*p* = 0.37 and *p* = 0.07 respectively) (Table 4). Additionally, the objective response rates (ORRs) were low for both groups—8% in the combined treatment and 4% in sorafenib alone. These findings indicate that dual therapy with sorafenib and erlotinib compared to sorafenib monotherapy provides no significant benefit in terms of overall survival or progression-free survival, and toxicity was exacerbated with this combination therapy [55].

Nogova and colleagues recently performed a phase I trial assessing the efficacy of sorafenib and everolimus dual therapy in patients with KRAS-mutated NSCLC adenocarcinoma, using CT scans and FDG-PET scans [56]. Initially, these researchers sought to find the ideal maximum tolerated dose (MTD) of dual everolimus + sorafenib therapy. The MTD in this study was reported as 7.5 mg everolimus and 800 mg sorafenib daily. In the extension of this trial, 17 patients received the MTD of this dual therapy, 13 of which had mutant KRAS NSCLC. A partial medical response (PMR) with an FDG-PET scan was determined at days 5 and 14 of the treatment (17% and 20%, respectively). However, when CT scans were performed several weeks later, zero of the patients showed an adequate partial response on the CT scan. Additionally, no significant correlation between the PET scan response in terms of PMR and PFS/OS was determined. The results of this study may suggest the poor efficacy of this combination therapy for KRAS-mutated NSCLC. However, given the nature of the phase I study design, the number of patients studied was certainly low compared to other clinical trials regarding the efficacy of sorafenib [56]. Further studies to improve treatment outcomes in KRAS-mutant NSCLC tumors are certainly suggested.

## 4. Conclusions and Future Perspectives

Numerous in vitro, in vivo, and clinical studies have indicated the potential therapeutic efficacy of sorafenib for use in NSCLC as a monotherapy or in combination with other agents. The synergistic behavior of sorafenib with a variety of other agents has been observed in numerous NSCLC cell lines and xenograft mice models, indicating a definitive potential for sorafenib combination therapies for the treatment of refractory NSCLC. The early BATTLE and MISSION clinical trials on sorafenib indicate statistically significant improvements in PFS and/or OS. However, follow-up studies aiming to understand how different NSCLC biomarkers, such as EGFR and KRAS mutations, impact sorafenib’s efficacy had variable findings regarding PFS and OS, which remains one of the limitations or factors in devising rational therapeutic combinations. Given the prevalence of sorafenib resistance in clinical practice, recent studies have focused on novel drug delivery mechanisms. It appears that novel mechanisms such as nanocarriers for drug delivery may improve the efficacy of the agent for patients with treatment-resistant NSCLC. Future directions could explore other anticancer drugs, targeted therapies, or immunotherapies in combination with sorafenib, considering variable factors such as adverse events and outcomes from prior treatments. In addition, ALK translocations have been recorded as important molecular targets in the treatment of NSCLC, therefore being important actionable genes for future studies [57]. Notably, drug delivery mechanisms such as extracellular vesicles, due to their ability to carry increased drug concentrations, are being explored for the enhanced efficacy of sorafenib for individuals with NSCLC.

## Figures and Tables

**Table 1:** Summary of combination indexes (CIs) from in vitro studies of dual-therapy approaches involving sorafenib in NSCLC cell lines.

Treatment(s)	Cell Line(s)	Drug Concentration	Combination Index (CI)
Sorafenib/gemcitabine [34]	A549	SF 0–32 µM/ GEM 0–64 µM	0.86
Sorafenib/pemetrexed [34]	A549	SF 0–32 µM/ PEM 0–32 µM	0.63
Sorafenib/gemcitabine [35]	A549	SF 5–10 µM/ GEM 5–10 µM	0.65
Sorafenib/betulinic acid [36]	A549, H358, A429	SF 1.3 µM/ BA 3 µM	0.749, 0.802, 0.497
Sorafenib/fingolimod [37]	A549	SF 2.5–10 µM/ FTY 2.5–10 µM	0.74
Sorafenib/CAI [38]	LLC	SF 0.5–10 µM/ CAI 1–20 µM	<1
Sorafenib/CAI [38]	A459	SF 0.5–10 µM/ CAI 1–20 µM	<1
Sorafenib/CAI [38]	H1975	SF 0.5–2.5 µM/ CAI 1–5 µM	<1
Sorafenib/CAI [38]	H1975	SF 5–10 µM/ CAI 10–20 µM	>1

GEM gemcitabine, PEM pemetrexed, BA betulinic acid, FTY fingolimod, CAI carboxyamidotriazole.

**Table 2:** **BATTLE trial median PFS and OS.** PFS progression-free survival, OS overall survival. * Indicates statistical significance between comparison group (*p* < 0.05).

Treatment(s)	Biomarker(s)	Median PFS	Median OS
Sorafenib [9,51]	Multiple/Not specific	2.83 months	8.48 months
Sorafenib [9,51]	EGFR FISH-negative	3.35 months *	Not reported
Sorafenib [9,51]	EGFR FISH-positive	1.84 months *	Not reported
High-conc Sorafenib Sensitivity Signature [9,51]	EGFR wild-type	3.61 months *	Not reported
Low-conc sorafenib sensitivity signature [9,51]	EGFR wild-type	1.84 months *	Not reported

**Table 3:** MISSION trial median PFS and OS.

Treatment(s)	Biomarker(s)	Median PFS	Median OS
Sorafenib [53]	EGFR-mutant	2.7 months *	13.9 months *
Placebo	EGFR-mutant	1.4 months *	6.5 months *
Sorafenib [53]	KRAS-mutant	2.6 months *	6.5 months
Placebo	KRAS-mutant	1.7 months *	5.1 months

* Indicates statistical significance between comparison group (*p* < 0.05).

**Table 4:** Additional findings of median PFS and OS.

Treatment(s)	Biomarker(s)	Median PFS	Median OS
Sorafenib [54]	KRAS-mutant	2.3 months	5.3 months
Sorafenib [55]	Multiple/Not specific	1.9 months	11.9 months
Sorafenib/Erlotinib [55]	Multiple/Not specific	3.1 months	8.9 months
Sorafenib/Everolimus [56]	KRAS-mutant	3.25 months	5.85 months

**Table 5:** Overall summary of clinical studies evaluating use of sorafenib for patients with different NSCLC biomarkers.

Treatment(s)	Biomarker(s)	Findings
Sorafenib [9,51]	EGFR wild-type vs. mutant	DCR is higher among patients with wild-type EGFR tumors vs mutant EGFR tumors. PFS not significant.
Sorafenib [9,51]	EGFR FISH-positive vs. FISH-negative	DCR and median PFS higher in EGFR FISH-negative tumors
Sorafenib sensitivity signature Low vs. high conc [9,51]	EGFR wild-type	PFS higher among pts treated with high-conc SSS. DCR not significant.
Sorafenib sensitivity signature [52]	KRAS mutation	Median PFS and OS not significant.
Sorafenib vs. placebo [53]	EGFR mutation	Median PFS and OS higher in patients with mutant EGFR who received sorafenib vs those with mutant EGFR who received placebo.
Sorafenib vs. placebo [53]	KRAS mutation	PFS increased in individuals treated with sorafenib. OS not significant.
Sorafenib [54]	KRAS mutation	The type of KRAS mutation did not significantly impact PFS or OS.
Sorafenib + Everolimus [56]	KRAS mutation	No significant correlation between PMR and PFS/OS

## Data Availability

Not applicable.

## Abbreviations

NSCLC	Non-small cell lung cancer
SCLC	Small cell lung cancer
VEGFR	Vascular endothelial growth factor receptor
PDGFR	Platelet-derived growth factor receptor
Flt-3	FMS-like tyrosine kinase 3
c-KIT	Type III receptor tyrosine kinase
RET	Rearranged during transfection
ID1	Inhibitor of differentiation 1
TKIs	Tyrosine kinase inhibitors
HCC	Hepatocellular carcinoma
HGF	Hepatocyte growth factor
EGFR	Epidermal growth factor receptor
ERK	Extracellular signal-regulated kinase
PI3K	Phosphatidylinositol 3-kinase
JAK	Janus Kinase
STAT	Signal transducers and activators of transcription
ETS1	ETS Proto-Oncogene 1
ACSL4	acyl-CoA synthetase long chain family member 4
CAFs	Cancer-associated fibroblasts
ccRCC	Clear cell renal cell carcinoma
NRF2	Nuclear factor erythroid 2-related factor 2
RUNX3	Runt-related transcription factor 3
CAI	Carboxyamidotriazole
mTOR	Mammalian target of rapamycin
DCR	Disease control rates
OS	Overall survival
PFS	Progression-free survival
SSS	Sorafenib sensitivity signature
ORRs	Objective response rates
MTD	Maximum tolerated dose
PMR	Partial medical response
